# A Qualitative Study on Voices of South African Learner Nurses on Occupational Health and Safety during Clinical Learning: Pre-COVID-19 Pandemic

**DOI:** 10.3390/nursrep13010010

**Published:** 2023-01-10

**Authors:** Livhuwani Muthelo, Tshepo Albert Ntho, Masenyani Oupa Mbombi, Thabo Arthur Phukubye, Mamare Adelaide Bopape, Tebogo Maria Mothiba

**Affiliations:** 1 Department of Nursing, University of Limpopo, Private Bag X1106, Sovenga, Polokwane 0727, South Africa; 2 Faculty of Health Science Executive Dean’s Office, University of Limpopo, Private Bag X1106, Sovenga, Polokwane 0727, South Africa

**Keywords:** occupational health and safety, clinical learning, learner nurses, nursing education institution

## Abstract

The demanding and complex training of learner nurses in clinical practices requires various occupational health and safety measures to curb occupational health hazards among learner nurses. This paper aimed to explore learner nurses’ experiences concerning occupational health and safety during clinical learning. A qualitative descriptive, contextual and exploratory design study was conducted. A total of 31 learner nurses were selected using purposive sampling. Data were collected by semi-structured interviews and analysed using Tesch’s open coding method. Two themes emerged from this study: “The experiences of learner nurses concerning their health and safety during clinical learning” and “The measures to be taken to promote occupational health and safety during clinical learning.” Highlighting challenges and measures to mitigate occupational health hazards among learner nurses in the South African context, Limpopo province, would be beneficial. The findings can assist stakeholders in developing specific precautionary guidelines for learner nurses. Effective and innovative occupational health and safety training programmes for learner nurses can be developed despite constrained environmental resources.

## 1. Introduction

The four-year comprehensive curriculum for training learner nurses is rigorous and complex as nursing education integrates two components, theoretical and clinical learning (CL). Theoretical elements equip student nurses with the information and perspective learned in the classroom. At the same time, clinical learning aims to improve the clinical competency and attributes of nursing among learner nurses [1]. Clinical practice allows learner nurses the opportunity to integrate theory into practice. CL of learner nurses is a fundamental component of nursing education and is essential in integrating theory into practice as prescribed by the South African Nursing Council regulations [2,3]. Despite various Occupational Health and Safety (OHS) laws and standard operating procedures and legislatures [4], learner nurses continue to be subjected to the same occupational health hazards as registered nurses during CL.

According to Amare, Tesfaye, Girmay and Gebreagziabher, occupational hazards are workplace issues that can increase the risk to an individual’s health and can be classified as either biological or non-biological [5]. Commonly, nurses encounter physical, chemical, ergonomic, and psychological hazards. For instance, a nurse who changes an obese patient’s position to prevent pressure ulcers may develop back pains. In addition, nurses are prone to psychological risks, such as stress and depression due to heavy workloads, shortage of staff, and inadequate equipment [2,5,6]. Subsequently, needle pricking and body fluids splashing on the skin and mucosa are occupational health hazards that commonly occur among learner nurses during CL, particularly when administering injectable medications and blood taking from the patient [6]. 

The World Nursing Report (2020) emphasized 10 key actions for improving nursing practice and training [7]. For instance, key issues 4, 6 and 7 stipulate that nursing training and practice should be aligned with national health priorities to adequately prepare nurses. Addressing the issues that impact clinical nurse learning, and providing support targeted to ensure occupational health and safety, must be prioritized and enforced [7]. The Sustainable Development Agenda to be realised by 2030, states under goals 3, 4, 8, and 16, respectively, to seek to ensure healthy lives and promote well-being for all at all ages. Equal access to vocational and tertiary education in advanced equitable and sustainable economic growth, employment, and decent work for all men and women across all fields and industries. Helping institutions to function according to legitimate laws, so that they do not succumb to arbitrary abuses of power [8,9,10]. There is a lack of conviction in achieving the 10 key actions for the future direction of the nursing workforce and specifically SDGs 3, 4, 8 and 16 due to the steady rise in occupational health hazards, particularly in the healthcare sector. The World Health Organization estimates the burden of diseases from occupational exposure to be 40% among healthcare providers worldwide [11]. The National Institute for Occupational Safety and Health claims that nursing is a high-risk profession [12,13]. This is evident in the extent of illness and injury sustained by nursing practitioners worldwide [13].

Although there is an interconnection of literature describing the OHS of healthcare workers, including registered nurses, only a few studies documented the occupational health hazards and safety among learner nurses during CL. These studies include the one conducted by Savci, Serbetci and Kilic, who emphasised that learner nurses are prone to occupational health hazards during the CL stage in healthcare settings [14]. Different studies reported poor compliance among learner nurses concerning OHS laws and standard operative procedures during CL [14,15]. This challenge is mainly perpetuated by a shortage of personal protective equipment, a lack of clinical mentorship, poor infrastructure, and a lack of knowledge concerning OHS laws and standard operating procedures among learner nurses during CL. Literature shows that in the United Kingdom, Europe, the Middle East, Canada, and Australia, learner nurses are commonly subjected to the same unpleasant occupational health injuries that registered nurses encounter [16,17]. As a result, these occupational health hazards among learner nurses hinder the efficiency of the CL. 

In Africa, Muthelo et al. and Mogale and Mogotlane argue that adherence to OHS laws is generally weak [4,18]. For instance, in Zambia, Chitimwango assessed nurses’ knowledge, attitudes and practices towards infection prevention and control at a selected tertiary hospital [19]. The study revealed that 66.2% (129) of all nursing categories could not apply infection prevention guidelines because they lacked enough time to comply with the infection prevention guidelines. Conversely, in Ghana, the conducted qualitative study by Alhassan and Poku revealed that the clinical practice of nursing staff in two psychiatric hospitals is risky and potentially unsafe given the poor health infrastructure situation in these health facilities [20]. The lack of guidelines for reporting occupational health hazards and seeking compensation for those harmed at work makes the issue worse. In South Africa, there is an increase in occupational health injuries among learner nurses [21]. Sehume’s findings reveal that selected public hospitals had an OHS nurse on-site, with some having an internal OHS policy that was communicated to workers [22]. Despite this, there was poor compliance with OHS policy, a lack of risk assessments, and inadequate provision of personal protective clothing [22]. 

Noting the above discussion, needle stick injuries are the most common way for blood-borne viruses such as HIV and hepatitis B and C to infiltrate the body [16,21]. According to Shil and Upashe [23], such infections pose significant occupational risks and threats to healthcare workers, mainly when crucial occupational health and safety policies are not effectively implemented and followed. Thus, nursing is regarded as a high-risk profession because of the nature of the work, as they frequently come into contact with blood and the risk of infectious diseases and needle prick injuries [11,12]. Globally, it is estimated that of approximately 35.7 million, 2 million healthcare practitioners are exposed to percutaneous exposures yearly [23]. The disproportionate burden that sub-Saharan Africa endures compared to developed countries is evident in the prevalence of HBV, HCV, and HIV among healthcare professionals who sustain sharp injuries. Makhado, l., Musekwa, Makhado, T.G. and Otsheleng, argue that in Africa, the prevalence of occupational exposure is approximately 92% among healthcare professionals [24]. For example, occupational exposure is responsible for 11.8% of HBV, 2.8 of HCV, and 5.1% of HIV infections in several African sub-regions, such as Botswana, Congo, Malawi, and South Africa.

Notably, in Africa, there seem to be very limited studies focused primarily on the learner nurses’ OHS compared to those of nurses in healthcare facilities. For example, in Eswatini, Gina, Rasweswe, and Moagi studies highlight that university learner nurses do not comply with handling and disposing of sharp objects after use and wearing personal protective clothing during CL, which exposes them to infectious diseases, such as hepatitis [25]. Personal protective gear includes wearing aprons, goggles, and masks to protect themselves from blood and bodily fluids. In the study conducted by Mabina, Morulane, Tong and Makhado in a Nursing Education Institution (NEI) in the North-West province among learner nurses concerning TB and HIV exposure, the majority reported that there was insufficient personal protective equipment in the clinical practices [15]. The shortage of personal protective clothing exposes learner nurses to occupational hazards and infectious diseases. 

In Limpopo province, learner nurses are not an exception to occupational health hazards. The researchers have observed with concern that the OHS of learner nurses from a selected NEI in the Limpopo Province is exposed to occupational health hazards and diseases during CL. This concern was observed during the researchers’ undergraduate tenure, when they were placed for CL at the South African Nursing Council (SANC) accredited clinical practices. According to the WHO, occupational diseases and injuries were responsible for the deaths of 1.9 million people in 2016, thus affecting future staff capacity [26]. Precautions to protect learner nurses’ health and safety are essential, including the risk assessment of the environment, proper sharp and waste disposal, and handling of patient care [27]. 

Experiential learning theory was adopted in this study to report on the voices of occupational health and safety among learner nurses during clinical learning. The theory is more applicable to this paper as it focuses on the environment in which learning takes place. According to Kolb, Boyatzis and Mainemelis [28] and Parchebafieh, Memarian and Vanaki [29], the learning environment consists of cognitive, social, cultural, affectional, emotional, motivational and academic factors. In the context of this study, the clinical environment covers all the conditions and stimuli that affect the clinical learning process. The learning environment comprises different factors such as learner nurses, clinical lecturers, clinical staff, academic activities, and the hospital environment, where learner nurses are exposed to different occupational hazards and risks such as infectious diseases and needle prick injuries.

The current study was conducted in rural areas where public hospitals are overburdened by disease with a shortage of staff and protective clothing and equipment. Ensuring the learner nurses’ safety has the potential of contributing to improving the capacity of healthcare workers and positively impacting the reduction of the shortage of nurses in the healthcare system. Furthermore, occupational safety for learner nurses could potentially contribute to increased clinical competency and practices with improved quality of healthcare services. It is for this reason that this study sought to determine the OHS of the learner nurses during CL in the Limpopo province. The findings of this study will guide policy and interventions in the research context and similar settings.

## 2. Materials and Methods

### 2.1. Design

COREQ Checklist was used to verify and complete the required sections of this qualitative manuscript [30]. A qualitative research approach with exploratory-descriptive and contextual research design was used in this study, as described by Polit and Beck [31]. The exploratory-descriptive research design enabled the researcher to understand the learner nurses’ perspectives of their experiences regarding their health and safety during CL at accredited clinical practices in Limpopo province [32]. On the other hand, the contextual design allowed the authors to obtain a picture of the natural situation as it occurred [32].

### 2.2. Setting

The paper sought to determine the health and safety of learner nurses during CL. The study was conducted at the selected NEI in the Limpopo Province of South Africa. This selected NEI is classified among the eight rural/historically disadvantaged institutions in South Africa [33]. This NEI is situated in the Capricorn District, approximately 32 kilometres from the eastern side of the capital city of Limpopo, Polokwane. It is accredited by the SANC to train learner nurses under Regulation 174 [34] and provides theory components in class and CL components at accredited clinical practices for training learner nurses.

### 2.3. Population and Sampling

A non-probability purposive sampling method was used to sample 31 learner nurses from the total population of 253 learner nurses, excluding the first-year-level learner nurses [31]. The target population was registered second, third and fourth-year learner nurses who had been attending CL at accredited clinical establishments [32]. This study excluded level one learner nurses from the selected NEI due to their lack of clinical exposure and limited scope of practice. The sample size of this study was determined by data saturation [35].

### 2.4. Data Collection

A female research assistant collected data in this study from September to November 2019 through one-on-one interviews using an interview guide (Table 1). The learner nurses were recruited from the class WhatsApp group. The details of the study, the aims and objectives, and how the interviews were to be conducted were fully explained to the participants. The research assistant outlined that participation in this study was voluntary and that the learner nurses could withdraw without fear of victimization. Each participant signed an informed consent form after being informed of their rights and that the interview would be audio recorded before data collection took place. Data were collected face-to-face in a secluded room to maintain privacy. The central question posed to all participants was: “Could you kindly explain the health and safety of learner nurses during clinical learning?” This was followed by probing questions as outlined in the interview guide. A voice recorder was used to capture verbal information, and field notes were written to capture non-verbal cues. Data were collected until data saturation was reached at participant number 31.

### 2.5. Data Analysis

The narrative data from interviews were analysed qualitatively using NVivo software version 12 and Tesch’s open coding method, as suggested by Creswell [36]. The authors repeatedly listened to the audio recording to ensure accurate transcription. The captured transcripts and field notes were loaded into Nvivo software for easy reading to enhance the overall sense of the entire data. The Nvivo software assisted the first two authors with coding the reference statements relevant to the study aim. The reference statements were further read to establish the new categories. The first two authors combined all the identified categories to form a list of sub-themes. Similar sub-themes were verified and re-coded, and by this stage, the authors determined the most descriptive wording for the proper naming of the themes. The themes and sub-themes were summarised and organised in table format for discussion with the last author. A meeting was held with the first and last two authors to validate the identified themes. The last author made a few technical arrangements to improve the meaning of the generated themes and sub-themes. 

### 2.6. Trustworthiness

Trustworthiness was ensured by applying Lincoln and Guba’s criteria of credibility, transferability, dependability and confirmability, as cited by Brink, Van der Walt and Van Rensburg as well as Korstjens and Moser [32,37]. The credibility and confirmability of the results were guaranteed by providing verbatim transcripts, voice recordings, and field notes for independent coding. The categories and sub-categories that emerged were compared with those of the researcher. Purposive sampling and a detailed description of the study methodology were used to ensure dependability and transferability in this study and to assist researchers who desire to conduct a similar study.

### 2.7. Ethical Consideration

Ethical clearance was obtained from the Turfloop Research Ethics Committee (TREC/300/2019: UG), and permission to conduct the study was obtained from the School of Health Sciences Director and the HoD of the Department of Nursing at the University of Limpopo. Participants were informed of the study’s aim and objectives. Additionally, learner nurses in the study were informed by a research assistant that their participation was voluntary and that they might withdraw their participation at any time without suffering any repercussions. Following that, a voluntary informed consent form was given to each participant who agreed to participate in the study, to sign. The participants’ identities were kept confidential and were not shared with anyone outside the research team. During data collection, learner nurses were given pseudonyms to help to achieve this, and the participants throughout the data collection process were referred to as Participants 1, 2, or 3, etcetera.

## 3. Results

### 3.1. Demographic Description

As depicted in Table 2 below a total of 31 participants were interviewed until data saturation was reached. Only 22 women and 9 men participated in the study, with their ages ranging between 18 and 25 years old. Jamieson, Harding, Withington and Hudson state that nursing is a predominantly female profession [38]. More males should be encouraged to make nursing their profession. In total, 7 learner nurses were in the second year of their study, 10 were in their third year, and 14 were in the final year of their undergraduate studies. All the participants were registered with the nursing education institution as learner nurses, training under Regulation 174 as SANC prescribes [34].

### 3.2. Themes and Sub-Themes Regarding the Occupational Safety of Learner Nurses

Table 3 below indicates the main themes that emerged from the experiences of the learner nurses during CL.

#### 3.2.1. Theme 1: Learner Nurses’ Health and Safety Experiences during Clinical Learning

The experience of learner nurses revealed personal protective equipment and supervision-related challenges concerning issues of their health and safety during CL. Learner nurses indicated that they are neither protected nor do they feel safe enough in various accredited clinical practices. Two sub-themes, namely, the inadequate provision of protective clothing and a lack of clinical mentoring by professional nurses, emerged within this theme and are discussed below.

##### Sub-Theme 1.1: The Inadequate Provision of Protective Clothing

The participants in this study revealed that inadequate protective clothing was provided to the learner nurses during CL. The following verbatim statements made by participants 07, 11, 27, and 30, respectively, reflected the extent and dire state of protective clothing of the learning nurses and how it had influenced their CL.

Participant 07: said: “*There are no gloves in some wards, such as the medical ward, which makes us susceptible to infections and patient bodily fluid*”.

Participant 11: said: “*I do not think we are safe. For example, in the gynaecology ward, we are instructed to do bed-making without wearing gloves which makes us unsafe*”.

Participant 27 said: “*According to my experience, nurses do not allow us to use sterile gloves when we do bed making. We are exposed to patients’ fluids*”.

Participant 30 said: “*With my experience from the medical ward particularly, we are made to nurse patients in side wards without any protection, those patients will usually have infectious conditions such as meningitis, and I was once made to feed the patient with meningitis without any protective clothing*”.

##### Sub-Theme 1.2: A Lack of Clinical Mentoring by Professional Nurses

Participants 01, 15 and 21 in this study reported that they were poorly supported and guided by professional nurses during their CL. According to the participants, the lack of support is aggravated by various reasons, such as a shortage of nursing staff, negative attitudes towards learner nurses and that nurses believed that supporting learner nurses at clinical practice during CL is the responsibility of nurse educators. The following extracts support this:

Participant 01 said: “*Sometimes we perform procedures without supervision, so we do not know if what we are doing is correct or not*”.

Participant 15 averred: “*Nurses were busy with patients only, and when we ask for help, they tell us that they cannot because they have many patients and there is a shortage, so we must assist one another*”.

Participant 21 also mentioned: “*I remember another nurse saying the government is not paying them to teach students but to provide care to patients, and that is the responsibility of our lecturers*”.

#### 3.2.2. Theme 2: The Measures to Be Taken to Promote Occupational Health and Safety during Clinical Learning

Data gathered demonstrated a need for promoting occupational health and safety during clinical learning by implementing various measures in the training institution and clinical facilities. The measures are discussed as five sub-themes, namely, the provision of voluntary vaccination against infectious diseases, proper education, management and provision of PPE, in-service training on OHS and infection control, and proper cleaning of the environment and maintenance of hospital infrastructure, including the availability of security guards in each ward. 

##### Sub-Theme 2.1: Provision of Voluntary Vaccination against Infectious Diseases

Participants in this study mentioned that voluntary vaccination of learner nurses against infectious diseases before their placement for CL will reduce occupational health hazards and diseases and promote healthy and safe clinical practices. The following quotations support what the participants said:

Participant 03 said: “*They must immunise us for all infectious diseases, such as hepatitis, before going to clinical learning*”.

Participant 09 said: “*Our department must make sure that we get vaccinated because they are aware of medical conditions which we are exposed to, and they should also make sure that they train nurses to make sure they keep us protected during clinical exposure at the hospital*”.

Participant 13 said: “*I think before we go to the hospital, the nursing department has to arrange with our university health centre so that students can go there and get immunised*”.

Participant 31 said: “*I think before being exposed to clinics and hospitals in the first year, we must all be immunized*”.

##### Sub-Theme 2.2: Proper Education, Management and Provision of Personal Protective Equipment

The learner nurses felt that the nurses should ensure that the students are provided with the necessary PPE and training on how to use it properly.

Participant 02 said: “*Nurses should ensure that students are provided with personal protective clothing every day when we arrive at the hospitals to prevent exposure to infections*”.

Participant 24 said: “*The hospital must ensure that there are protective clothing and medical equipment that is needed for the safety of nurses and students as we are nursing patients with complications*”.

Participant 29 said: “*They must educate us about the importance of using PPE and how and when to use it. For example, should we wear sterile gloves when monitoring blood glucose?”*

##### Sub-Theme 2.3: In-Service Training on Occupational Health and Safety and Infection Control

According to participants in this study, learner nurses require adequate in-service training on OHS and infection prevention and control. Participants believed:

Participant 06 stated: “*I think in the hospital or clinic they should implement in-service training where they delegate one professional nurse to teach the student nurses on measures to protect themselves during practicals, they can teach them on the waste disposal which can be done on two weeks or monthly*”.

Participant 16 shared: “*Nurses should teach us how to safely perform aseptic procedures to ensure the safety of the patients and themselves as the nurses*”.

Participant 25 averred: “*I think the manager has to educate the students on how to maintain safety. For example, if a nurse has been needle pricked, they must make sure that they educate them on how to implement steps that are necessary to prevent being infected by diseases such as HIV. They must also educate them about the importance of wearing a PPE, and the PPE should be available in the clinical area*”.

##### Sub-Theme 2.4: Proper Cleaning of the Environment and Maintenance of Hospital Infrastructure

Proper maintenance of the clinical facilities is one of the measures that can be taken to promote learner nurses’ OHS during clinical learning. The following utterances confirm this: 

Participant 06 said: “*Department should ensure that the hospital’s maintenance is in good condition. For instance, broken wires and plugs that are not working are correctly placed*”.

Participant 04 said: “*Nurses should make sure that students are safe during clinical exposure, where they prevent needle pricks by proper disposal of sharps*”.

Participant 08 said: “*Honestly, the department must do something, I mean, water is a problem in this hospital, and sometimes we spend the whole day without water. So we know that, as a student, it is important to carry your bottle of water*”.

##### Sub-Theme 2.5: The Availability of Security Guards in Every Ward

According to the participants of this study, security guards should be available in each ward to ensure the safety of learner nurses during CL. The following extracts support this:

Participant 04 said: “*There should be security guards at each ward to prevent attacks, and they should search visitors before entry*”.

Participant 17 said: “*Patients sometimes abscond, and it became challenging for nurses to trace them because many people go in and out freely without proper control*”.

Participant 22 said: “*It is not safe, imagine people who fought at the tavern with a gang group, and that person is admitted. That gang group can come to the hospital to finish him up, and what about us students?*”

## 4. Discussion

The paper presented the voices of learner nurses concerning occupational health and safety during clinical learning using experiential learning theory. The following themes were used to determine the OHS of the learner nurses during CL in the Limpopo province.

### 4.1. Demographic Profile

A total of 31 people were interviewed until data saturation was reached. The study participants were aged between 18 and 25 of which 22 were women and 9 were men. Females still dominate the nursing profession [38]. Therefore, recruitment of male nurses is still needed in the nursing profession. In total, 7 student nurses were in their second year of study, 10 were in their third year, and 14 were in their final year of undergraduate studies. All participants were registered with the nursing education institution as learner nurses pursuing an undergraduate nursing programme qualification under SANC Regulation 174.

### 4.2. Experiences of Learner Nurses concerning Their Health and Safety during Clinical Learning

The experiential learning theory demonstrated that the environment in which clinical learning takes place is dominated by the inadequacy of personal protective clothing and equipment provided, which are one of the main barriers to effective CL among learner nurses. The study findings have revealed that although the CL of learner nurses is crucial, learner nurses are exposed to occupational health hazards due to inadequate personal protective clothing and equipment provided during their CL. Globally, it has been documented that learner nurses are not provided with protective equipment against occupational health hazards. Case in point, in the United Kingdom, the literature highlights that learner nurses struggle to obtain adequate personal protective equipment [39]. In addition, the effectiveness of the CL and the provision of health care to the patient is impeded by various challenges, which include a lack of protective clothing and equipment for the learner nurses [40,41]. From the above discussion, it is evident that inadequate provision of personal protective equipment poses severe occupational health hazards to learner nurses during CL. Addressing this challenge, will reduce occupational health hazards and promote a facilitative CL amongst learner nurses.

The experiential learning theory identified clinical staff members as one of the factors that have an impact on the learning environment. Registered nurses in this study were reported to be unsupportive, which hindered the effectiveness of CL among learner nurses. The study conducted by Ntho, Pienaar and Sehularo, who explored the challenges learner nurses experience concerning mentorship during CL, confirms this study’s results [42]. These authors, in their study, found that mentors in clinical practices were unapproachable, autocratic, and had a disparaging attitude towards mentoring the learner nurses. Furthermore, learner nurses reported that a lack of support and guidance was perpetuated by a shortage of nurses and the nurses’ attitude toward supporting learner nurses. The clinical competence of learner nurses is influenced by factors such as a lack of support, the attitude of the nurses towards their learning, and the shortage of nurses within the clinical facilities. The clinical competence of learner nurses is influenced by various factors that call for the support of all stakeholders during CL. Similarly, the research on learner nurses’ clinical support in the management of TB and HIV patients in a primary healthcare setting highlighted the confusion among learner nurses and the fear of becoming infected during CL, which was closely linked to poor or inadequate preceptorship [43]. There is a strong need to augment the clinical mentoring of learner nurses during CL. The reinforced clinical mentoring will alleviate the fear among the learner nurses that are said to be exacerbating occupational health hazards, and ultimately this will facilitate the transition of learner nurses in acquiring clinical competence [3]. The study findings emphasize the role of registered nurses and nurse educators in supervising, supporting and mentoring learner nurses during CL.

### 4.3. Measures to Be Taken to Promote Occupational Health and Safety during Clinical Learning

Despite the challenges cited above, participants suggested measures and practices that may be used to promote OHS among learner nurses during CL. Participants averred that NEIs must provide voluntary vaccinations against infectious diseases for all learner nurses during their first year of study and before CL placement. Centres for Disease Control and Prevention strongly recommend that all health workers, including learner nurses, receive appropriate vaccines to reduce the chance of contracting or spreading vaccine-preventable diseases as they are at risk for exposure to severe and sometimes deadly diseases [44]. If adequately implemented, a voluntary vaccination campaign and receiving preventive therapies against infectious diseases among learner nurses could help to curb vaccine-preventable diseases during CL [15].

The current study findings commend the role of PPE in preventing occupational health hazards and diseases among learner nurses during CL. Participants in this study indicated that proper education on how to use PPE efficiently as well as its appropriate handling and distribution among registered nurses and learner nurses might help clinical practices to minimise the shortage of PPE and prevent occupational health hazards and diseases, thus promoting effective CL. The current study findings are similar to those reported in the existing literature. For example, Makhado and Seekane recommend the compulsory use of PPE in clinical practices among healthcare workers and learner nurses [21]. These authors further put forward that an independent person or team should be appointed to monitor the use of PPE among learner nurses and healthcare workers to reduce the incidences of occupational exposure. The study findings highlight the gap in integrating theory into practice during CL among learner nurses. Constant awareness of PPE and addressing these challenges will encourage compliance with OHS legislature and standard operating procedures and reduce occupational health hazards among learner nurses during CL.

According to Brown, Munro and Rogers, a comprehensive understanding of infection prevention and control is essential for learner nurses to protect themselves, patients, colleagues and the general public from the transmission of infectious diseases [45]. Knowledge of OHS, infection prevention, and control should be promoted, strengthened and emphasised among learner nurses [15]. An examination of the literature suggests various recommendations to promote adherence to standard operative procedures of OHS among learner nurses during CL. These recommendations include in-service training, clinical mentoring and reinforcing the undergraduate nursing education curriculum [15,39,42,43]. Therefore, this study’s findings provide the baseline for developing ongoing in-service training on OHS and infection control for learner nurses.

The study findings revealed that learner nurses view proper cleaning of the environment and maintenance of clinical practices as necessary measures to prevent occupational health hazards during their CL. Similarly, in a qualitative study conducted at 27 hospitals in South Africa, most healthcare workers felt that poor infrastructure is a risk factor for the transmission of airborne diseases, such as tuberculosis [46]. Literature corroborates the results of this study that dilapidated or poor infrastructure remains a challenge among many public hospitals in Limpopo province. For example, in Limpopo, a study by Netshisaulu, Malelelo-Ndou and Ramathuba indicate that most clinical practices were developed by missionaries and infrastructure is aged and in a dilapidated state. These results imply that intervention is required to protect learner nurses, healthcare workers and patients and ensure safer clinical practices [47]. 

Participants in this study expressed concern that they do not feel safe during their CL because anyone has free access to the ward. Participants stated that safety in a public healthcare facility is a challenge. Violence against healthcare workers, including learner nurses, is not new and has been a pandemic [48,49]. Although there is an interconnection of literature describing violence in the workplace, not many studies documented the safety and violent incidents against learner nurses during CL. The descriptive cross-sectional survey by Majola investigating violence against student nurses by patients and their relatives in public hospitals in KwaZulu-Natal confirms the findings of this study [50]. One of the study’s most striking findings is that many learner nurses are the targets of verbal abuse, bullying, and intimidation [50]. According to Manyisa, a lack of safety in selected public hospitals in South Africa is a concern, as a high rate of crime and violence in the form of assaults are common in clinical practices [51]. In addition, various violent incidents were widely documented worldwide. For instance, according to a WHO report, in 2021 alone, over 800 confirmed attacks on healthcare facilities took place worldwide [52]. The findings of this study suggest that a lack of comfort, security and safety during CL hampers the accomplishment of clinical competence among learner nurses.

### 4.4. Limitation of the Study

This study was conducted only in one NEI in Limpopo province. This limits the generalisability of the findings to other NEIs. However, the findings and recommendations of this research can be applied to other NEIs in South Africa. 

### 4.5. Recommendations

Ongoing in-service training on OHS and infection prevention and control for learner nurses is strongly recommended based on the study results. The selected NEI that provides training to learner nurses in Limpopo Province should develop specific precautionary guidelines to safeguard learner nurses’ health and safety and facilitate effective CL. Registered nurses in clinical practices and nursing educators of the NEI should mentor learner nurses during CL. Further research is recommended on developing scientific solid-based learner nurses’ OHS training programmes in the Limpopo province.

## 5. Conclusions

Despite the availability of OHS legislation or standard compliance with various clinical practices, the study findings revealed that learner nurses are exposed to severe occupational health hazards during CL due to inadequate provision of personal protective equipment and clinical mentoring. Nevertheless, the CL of learner nurses is a vital component of nursing education in fostering clinical competency amongst learner nurses.

## Figures and Tables

**Table 1:** Interview guide.

Central question	
	Could you kindly explain the health and safety of learner nurses during clinical learning?
Probing questions	
	What are your views on the health and safety of learner nurses during clinical learning? What factors contribute to an unsafe environment during clinical learning? What health and safety challenges have you experienced during clinical learning? Which measures can be taken to maintain the health and safety of learner nurses during clinical learning?

**Table 2:** Demographic profile of learner nurses.

Demographic Variables	Population, *n* (%)
Gender	
Male	9 (29%)
Female	22 (71%)
Age	
18–21	20 (65%)
22–25	11 (35%)
>26 years	0 (0%)
Years of study	
Second level	7 (23%)
Third level	10 (32%)
Fourth level	14 (45%)
Total	31 (100%)

**Table 3:** Themes and sub-themes regarding the occupational safety of learner nurses.

Themes	Sub-Themes
1. Learner nurses’ health and safety experiences during clinical learning.	The inadequate provision of protective clothing. A lack of clinical mentoring by professional nurses.
2. The measures to be taken to promote occupational health and safety during clinical learning.	The provision of voluntary vaccination against infectious diseases. Proper education, management and provision of personal protective equipment. In-service training on occupational health and safety and infection control. Proper cleaning of the environment and maintenance of hospital infrastructure. The availability of security guards in each ward.

## Data Availability

The data presented in this study are available on request from the corresponding author. Data are not made publicly available due to the privacy terms signed by the participants in the informed consent forms.
